# High‐throughput droplet microfluidics screening platform for selecting fast‐growing and high lipid‐producing microalgae from a mutant library

**DOI:** 10.1002/pld3.11

**Published:** 2017-09-27

**Authors:** Hyun Soo Kim, Shih‐Chi Hsu, Song‐I Han, Hem R. Thapa, Adrian R. Guzman, Daniel R. Browne, Mehmet Tatli, Timothy P. Devarenne, David B. Stern, Arum Han

**Affiliations:** ^1^ Department of Electrical and Computer Engineering Texas A&M University College Station TX USA; ^2^ Korea Institute of Machinery and Materials Daegu Research Center for Medical Devices and Rehabilitation Daegu South Korea; ^3^ Boyce Thompson Institute Ithaca NY USA; ^4^ Department of Biochemistry and Biophysics Texas A&M University College Station TX USA; ^5^ Department of Biomedical Engineering Texas A&M University College Station TX USA

**Keywords:** biofuel, *Chlamydomonas reinhardtii*, droplet microfluidics, high‐throughput screening, microalgae, mutant library screening, single‐cell analysis

## Abstract

Biofuels derived from microalgal lipids have demonstrated a promising potential as future renewable bioenergy. However, the production costs for microalgae‐based biofuels are not economically competitive, and one strategy to overcome this limitation is to develop better‐performing microalgal strains that have faster growth and higher lipid content through genetic screening and metabolic engineering. In this work, we present a high‐throughput droplet microfluidics‐based screening platform capable of analyzing growth and lipid content in populations derived from single cells of a randomly mutated microalgal library to identify and sort variants that exhibit the desired traits such as higher growth rate and increased lipid content. By encapsulating single cells into water‐in‐oil emulsion droplets, each variant was separately cultured inside an individual droplet that functioned as an independent bioreactor. In conjunction with an on‐chip fluorescent lipid staining process within droplets, microalgal growth and lipid content were characterized by measuring chlorophyll and BODIPY fluorescence intensities through an integrated optical detection system in a flow‐through manner. Droplets containing cells with higher growth and lipid content were selectively retrieved and further analyzed off‐chip. The growth and lipid content screening capabilities of the developed platform were successfully demonstrated by first carrying out proof‐of‐concept screening using known *Chlamydomonas reinhardtii* mutants. The platform was then utilized to screen an ethyl methanesulfonate (EMS)‐mutated *C. reinhardtii* population, where eight potential mutants showing faster growth and higher lipid content were selected from 200,000 examined samples, demonstrating the capability of the platform as a high‐throughput screening tool for microalgal biofuel development.

## 1 INTRODUCTION

Microalgae have received considerable interest as a promising renewable bioproduction platform due to their capability of utilizing sunlight and atmospheric CO_2_ for producing a wide range of biomolecules, which can be converted into biofuel, food, feed, and other co‐products (Chisti, 2013; Georgianna & Mayfield, 2012; Han, Hou, Li, Kim, & de Figueiredo, 2013; Han, Jin, Tu, & Wu, 2015; Mata, Martins, & Caetano, 2010; Scott et al., 2010). With regard to biofuel feedstocks, microalgae have competitive advantages such as faster growth, higher lipid yield, and less competition with food supply and land usage compared to oil‐producing crops such as corn, soybean, and sugarcane that are currently being used for ethanol, biodiesel, or second‐generation fuel production (Mata et al., 2010; Scott et al., 2010). Despite this tremendous potential, microalgal biofuel production is neither economically competitive nor fully sustainable, and significant improvements are required for commercial viability (Chisti, 2013; Georgianna & Mayfield, 2012; Kim, Weiss, Thapa, Devarenne, & Han, 2014).

Developing microalgal strains with faster growth rates and/or higher lipid content through genetic improvement and metabolic engineering is an important component to overcoming existing barriers to commercial development (Chisti, 2013; Georgianna & Mayfield, 2012; Ghosh et al., 2016; Hlavova, Turoczy, & Bisova, 2015; Kim, Devarenne, & Han, 2015; Kim, Guzman, Thapa, Devarenne, & Han, 2016; Scranton, Ostrand, Fields, & Mayfield, 2015). Approaches relying on genetic transformation and random mutagenesis have been widely utilized and contributed to obtaining better‐performing microalgal strains. For example, several *Chlamydomonas reinhardtii* starch synthesis mutants, including *sta6*, which is used in the present work, exhibited increased lipid accumulation (Siaut et al., 2011; Work et al., 2010). Successful screening for such mutants requires single‐cell resolution measurement of the attributes of interest from large mutant populations, as each member of the population potentially has a unique profile. However, conventional screening where diluted pools of cells are cultured on media plates and transferred into separate microplate wells for the characterization of individuals, is too laborious and time‐consuming to employ at large scale.

Recently, fluorescence‐activated cell sorting (FACS) has been applied as a high‐throughput quantitative method of screening microalgal mutants. FACS in combination with fluorescent lipid staining has successfully detected and isolated high lipid‐producing mutants (Terashima, Freeman, Jinkerson, & Jonikas, 2015; Xie et al., 2014). However, while FACS‐based screening is efficient for the analysis of lipid content, other important traits such as cell division rates cannot be examined. Also, FACS is generally found in core facilities due to expensive equipment and operational expertise required. Therefore, we sought to develop an efficient and low‐cost single‐cell screening strategy that could analyze both lipid content and cell division rate.

Droplet microfluidics‐based systems have shown the capability to outperform conventional biological assays by conducting complex and highly reproducible screening at extremely high throughput (Guo, Rotem, Heyman, & Weitz, 2012; Lagus & Edd, 2013; Rakszewska, Tel, Chokkalingam, & Huck, 2014). Through a water‐in‐oil emulsion process, thousands of monodisperse droplets can be generated each second, with each aqueous droplet suspended in carrier oil comprising an independent bioreactor (Bardin, Kendall, Dayton, & Lee, 2013). These droplets, each encapsulating one or more cells, can be individually manipulated and analyzed (Han, Kim, & Han, 2017). Droplet microfluidics‐based systems have been successfully utilized in a broad range of applications such as drug discovery, synthesis of biomolecules, diagnostic testing, enzyme activity, and directed protein evolution (Hua et al., 2010; Jakiela, Kaminski, Cybulski, Weibel, & Garstecki, 2013; Kim et al., 2017; Mazutis et al., 2009; Ostafe, Prodanovic, Lloyd Ung, Weitz, & Fischer, 2014; Seiffert & Weitz, 2010; Sjostrom et al., 2014; Wang et al., 2014).

Here, we present a high‐throughput droplet microfluidics‐based screening platform that allows for analyzing growth and lipid content from microalgal cells, followed by selective retrieval and off‐chip analysis of samples showing the enhanced traits. The platform was initially used for proof‐of‐concept screenings utilizing mixtures of known fast‐ and slow‐growing, or high‐ and low‐lipid content strains. Then, we screened 200,000 individual variants from an ethyl methanesulfonate (EMS)‐mutagenized population, and found eight variants showing faster growth and higher lipid content. This platform enables, for the first time, the entire screening procedure to be conducted on a single microfluidic platform.

## 2 RESULTS

### 2.1 Design of the screening platform

**Figure 1:**
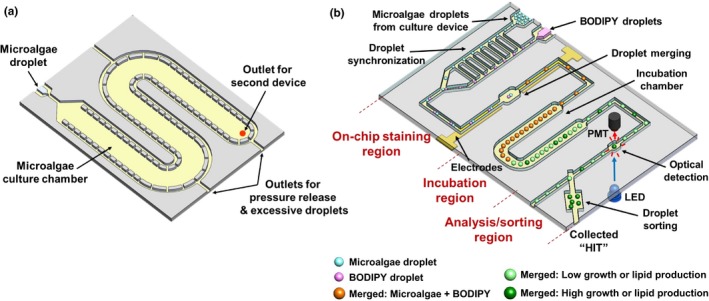
Illustration of the two‐module high‐throughput droplet microfluidics‐based microalgae screening platform. (a) Droplet generation/culture module. (b) Droplet staining/analysis/sorting module

The platform is composed of two interconnected micromodules: a droplet generation/culture module and a droplet staining/analysis/sorting module (Figure 1; Fig. S1). The overall operation of the system is visualized in Movie S1. First, in the droplet generation/culture module (Figure 1a; Fig. S1a), single microalgal cells suspended in culture media are individually encapsulated into droplets through a T‐junction droplet generator. This droplet generator consists of a 200‐μm‐wide channel for carrier oil and a perpendicular 160‐μm‐wide orifice channel for culture media (Fig. S1c), where the faster oil flow shears the cell‐suspended culture media to generate 240‐μm‐diameter droplets (volume: 7.24 nl: a single *C. reinhardtii* cell in a droplet is equivalent to a concentration of 1.38 × 10^5^ cells/ml). The droplets then flow into a downstream culture chamber (total volume: 70 μl) and were typically incubated for 1.5–4 days to achieve growth inside the droplets (Fig. S1e). An array of pillars separated by 40‐μm gaps was utilized along the side walls of the culture chamber to allow continuous carrier oil flow while all droplets remained trapped inside, resulting in a highly packed droplet array inside the culture chamber (Fig. S1e).

After the on‐chip culture step, the incubated droplets were then transferred to the droplet staining/analysis/sorting module through a tubing connection. This module is comprised of three functional parts: an on‐chip droplet staining region, a droplet incubation region, and a droplet analysis/sorting region (Figure 1b; Fig. S1b). The on‐chip droplet staining region follows the conceptual framework of our previous design (Kim et al., 2016), where droplets containing the lipid‐staining fluorescent dye (BODIPY) are merged with the droplets containing cultured cells to enable the quantification of lipid content within the droplets. First, 240‐μm‐diameter droplets containing BODIPY dissolved in 2% dimethyl sulfoxide (DMSO) in culture media were generated and synchronized with the cell‐containing droplets (released from the culture chamber) through a railroad‐like structure as previously reported (Ahn, Lee, Lee, Panchapakesan, & Oh, 2011; Kim et al., 2016), which enabled one‐to‐one droplet pairing (Fig. S1d). By applying an electric field with an integrated electrode pair, these synchronized droplets were merged into a single droplet inside a merging chamber (Fig. S1f) (Guzman, Kim, de Figueiredo, & Han, 2015). This exposed the encapsulated microalgae to BODIPY, fluorescently staining lipids inside the cells. The merged droplets then passed through the droplet incubation chamber for complete lipid staining (Fig. S1g; droplet traveling time from merging until reaching the detection zone = 11 min = incubation time).

Growth and lipid content were quantified through an integrated optical detection system while the incubated droplets flowed through an optical detection channel in the analysis/sorting region (Figs. S1h and S2). The analysis/sorting region was designed to have a shallower channel (height: 100 μm) than other parts of the platform (height: 160 μm) for placing most cells within the focal plane of the optical detector. In addition, the optical detection channel is 400 μm wide so the entire droplet is within the detection zone, enabling all cells inside a droplet to be detected as a whole. Additional oil injection through another inlet in this region created additional spacing between droplets before droplets entered the detection zone, so that only one droplet is measured at a time (Fig. S1h). A blue LED was used to simultaneously excite both chlorophyll autofluorescence (growth indicator, red emission) and BODIPY fluorescence (lipid indicator, green emission). Emitted light from a droplet was split through a bandpass filter and then detected by two compact photomultiplier tubes (PMTs; Fig. S2a). Based on this simultaneous measurement, droplets showing higher chlorophyll (i.e., enhanced growth) and higher BODIPY (i.e., enhanced lipid content) signals were sorted for off‐chip analysis. For this purpose, a hydrodynamic droplet sorting method was utilized where transient oil injection from an oil reservoir caused the flow to change in the main droplet channel, pushing the target droplet into a collection chamber. The entire operation of the system was controlled through a LabView interface.

### 2.2 Platform characterization: droplet generation, synchronization, merging, analysis, and sorting

**Figure 2:**
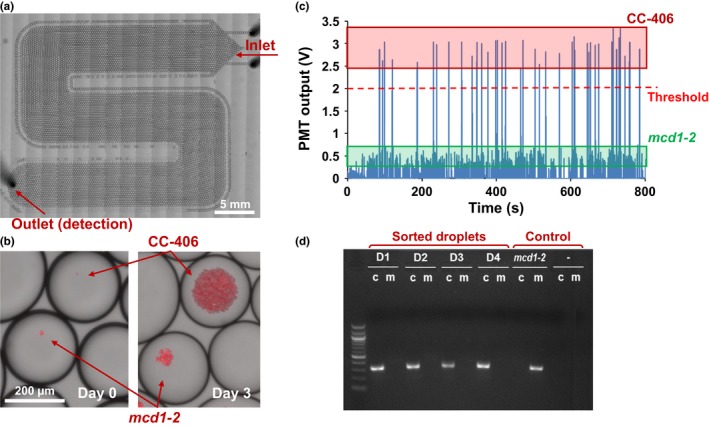
Growth screening using *C. reinhardtii* strains CC‐406 and *mcd1‐2*. (a) Culture chamber example housing 8,000 droplets. (b) Enlarged view of droplets showing different growth rate between CC‐406 and *mcd1‐2* cells in 100% acetate. (c) Chlorophyll autofluorescence detection from CC‐406 and *mcd1‐2* cells encapsulated in droplets. (d) Genotyping PCR results confirming the sorted droplets with higher optical output contained CC‐406 cells. D1‐4: samples derived from four sorted droplets; *mcd1‐2*: negative control; ‐: DNA‐free control; c: CC‐406; m: *mcd1‐2*

Droplets containing single cells were generated using a carrier oil flow rate of 600 μl/hr and a cell suspension flow rate of 250 μl/hr. The number of cells encapsulated in droplets follows a Poisson distribution, and ~25% of the generated droplets contained a single cell (Fig. S3). These droplets were flown into the downstream culture chamber, where about 8,000 droplets were stored without merging during the multiday culture period (Figure 2a). Droplets of the same size containing BODIPY were produced by flowing oil and BODIPY solutions at rates of 110 and 40 μl/hr (combined flow rate: 150 μl/hr), respectively. By reflowing the cultured droplets into the synchronization channel at 150 μl/hr along with the BODIPY droplets, the two trains of droplets formed one‐to‐one pairs. By applying a 200 V square wave (10 kHz) to the merging electrodes, more than 95% of the paired droplets were successfully merged. Occasionally, more than two droplets merged (<5%), but as these droplets typically divide into several smaller droplets in the droplet analysis/sorting region due to the high flow rate of spacing carrier oil (900 μl/hr), optical detection data from these droplets could be easily excluded.

As the emission spectrum of Nile red overlaps with that of chlorophyll autofluorescence, we used BODIPY, which has an emission spectrum outside that of chlorophyll autofluorescence, to allow simultaneous analysis of the number of cells within a droplet for a cell division proxy and lipid content (Fig. S2b,c). As efficient BODIPY lipid staining requires DMSO, which can be toxic to cells, various concentrations of DMSO were tested on the wild‐type strain CC‐406 and the *sta6* mutant. As shown in Fig. S4a, cells treated with less than 2.5% DMSO during on‐chip staining remained viable. However, as *C. reinhardtii* cells treated with 2.5% DMSO showed slower growth (Kim et al., 2016), 1% DMSO was used for subsequent on‐chip staining. The amount of BODIPY was further optimized with 1% DMSO, and a concentration of 500 μg/ml with a 10‐min incubation was chosen, which shows around 97% staining efficiency compared to the off‐chip stained control sample (Fig. S4b).

In the optical detection chamber, single droplets were measured one at a time at a rate of 5 droplets/s. The optical detection system was characterized by measuring total chlorophyll autofluorescence from droplets containing different numbers of cells. A strong correlation (*R* ^2^ = 0.9908) between fluorescence intensity and cell number was obtained, with a sensitivity as low as 2–3 cells (Fig. S5). Nonuniform cell size and metabolic state may result in chlorophyll autofluorescence intensity variations for each cell within a droplet, and may be the cause of the slight optical measurement differences when the number of cells in given droplets was all the same. This may have limited the detection sensitivity to 2‐3 cells per droplet. When the optical output from any droplet showed a value higher than a set threshold, a pulse of oil was injected into the droplet sorting channel, which diverted the flow from the waste outlet into a collection outlet. This directs only the target droplets into the collection chamber (Fig. S6).

### 2.3 Screening for more rapidly dividing microalgal clones

To demonstrate the platform's ability to distinguish growth/division rates, two *C. reinhardtii* strains CC‐406 (*cw15*) and *mcd1‐2* were used (Davies & Plaskitt, 1971; Drager, Girard‐bascou, Choquet, Kindle, & Stern, 1998). The cell wall‐deficient strain CC‐406 has a wild‐type growth rate (Davies & Plaskitt, 1971) and was used as a fast‐growing strain. Strain *mcd1‐2* is isogenic to CC‐406 except for a point mutation in *MCD1*, which is required for stability of the chloroplast *petD* transcript (Drager et al., 1998). As a consequence, *mcd1‐2* is nonphotosynthetic and requires acetate for growth (Drager et al., 1998). We grew CC‐406 and *mcd1‐2* under limited acetate concentrations to cause relatively slow growth of the latter strain. To do so, cell densities were monitored inside droplets with either 0%, 40%, or 100% of the usual acetate concentration in TAP medium, corresponding to 0, 6.96, and 17.4 mM, respectively. The growth of CC‐406 was similar in all three media, while *mcd1‐2* showed highest growth at 100% acetate, and failed to grow without acetate (Fig. S7). Because CC‐406 grew six to seven times faster than *mcd1‐2* in 100% acetate media during the three‐day culture period, this medium was used in the proof‐of‐concept experiment. CC‐406 grows faster because under the given conditions both photoautotrophic and heterotrophic growth mechanisms are utilized.

A mixture of CC‐406 and *mcd1‐2* at a cell ratio of 1:10 was used to test the platform by first generating droplets encapsulating single cells, which were incubated in the downstream culture chamber for 3 days (Figure 2a,b). In an analysis of 461 droplets, a bimodal pattern of chlorophyll fluorescence was observed, with 44 droplets above the threshold and 417 below, close to the initial 1:10 ratio (Figure 2c). We presumed that the 44 droplets harbored CC‐406 cells and that the 417 droplets harbored *mcd1‐2* cells, with the latter exhibiting five to six times less fluorescence intensity (Figure 2c). The observed PMT outputs were consistent with those obtained from separate experiments where CC‐406 and *mcd1‐2* were analyzed independently.

To validate the assumed genotypes, droplets displaying above‐threshold chlorophyll autofluorescence intensity and thus assumed to be CC‐406 were sorted to an off‐chip collection chamber and grown on solid agar media. All plated droplets gave rise to colonies, and 12 were randomly selected for genotyping. Primers were designed to distinguish the wild‐type *MCD1* gene in CC‐406 from the nonsense point mutation in *mcd1‐2*. As shown in Figure 2d, all colonies were confirmed to be CC‐406 and not *mcd1‐2*. This result demonstrated that the device has the capability to distinguish and select clones that show more rapid growth.

### 2.4 Screening for microalgal clones producing higher lipid content

Lipid detection was optimized by comparing *C. reinhardtii* strains CC‐406 and CC‐4333. CC‐4333 has a *cw15 sta6‐1* genotype, will be referred to as *sta6*, and is blocked in starch synthesis due to a mutation affecting ADP glucose phosphorylase (Zabawinski et al., 2001). This leads to lipid accumulation in *sta6* to a higher level than that of CC‐406 under nitrogen deprivation (Li, Han, Hu, Sommerfeld, & Hu, 2010; Wang, Ullrich, Joo, Waffenschmidt, & Goodenough, 2009; Work et al., 2010). These high and low lipid‐producing strains were first grown under 5%, 10%, 20%, 30%, and 100% nitrogen conditions (corresponding to 0.37, 0.75, 1.50, 2.24, and 7.48 mM nitrogen in TAP medium, respectively) to compare growth rates, as well as to determine the nitrogen level that would lead to nitrogen starvation and thus lipid accumulation after 2 days of growth. While cell division rates were highly responsive to nitrogen limitation, the responses under all nitrogen concentrations except for 10% were equivalent between the two strains (Fig. S8). This indicates that lipid content could be quantified using BODIPY without having to consider chlorophyll autofluorescence intensity as a proxy for growth rate. A nitrogen concentration of 30% was used in the subsequent screening experiment as growth saturated inside droplets after one to two days of culture (Fig. S8).

**Figure 3:**
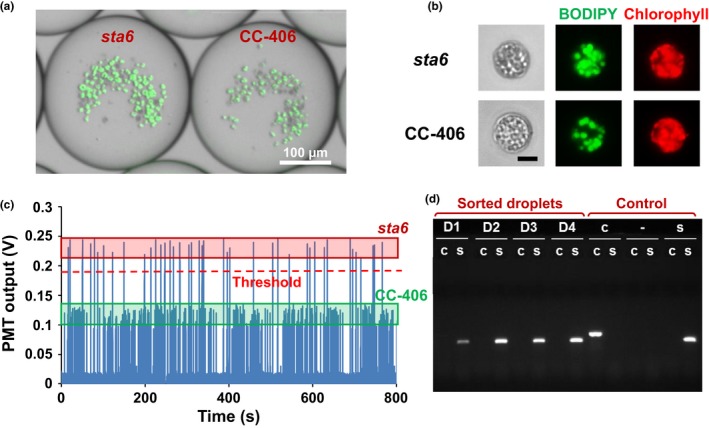
Lipid content screening using CC‐406 and *sta6*. (a) Microscopic images of droplets after on‐chip BODIPY staining after 4 days of culture. (b) Enlarged view of CC‐406 and *sta6* cells inside droplets. Scale bar = 5 μm. (c) Characterization of lipid content through BODIPY fluorescence detection illustrating the difference in optical signals from droplets harboring CC‐406 vs. *sta6*. (d) Genotyping PCR results confirming the sorted droplets with higher optical output contained *sta6* cells. D1‐4: samples recovered from four sorted droplets; ‐: DNA‐free control; c: CC‐406; s: *sta6*

A CC‐406:*sta6* cell ratio of 10:1 was introduced into the platform and cultured for 4 days in droplets: 2 days to proliferate and another 2 days for lipid accumulation. The droplets were then optically analyzed following on‐chip BODIPY staining. Figure 3a shows a microscopic image of droplets after on‐chip staining, where the droplet containing presumed *sta6* cells displayed 2.2‐fold higher fluorescence intensity than the droplet containing presumed CC‐406 cells. This difference in lipid content is also evident in Figure 3b, which shows an enlarged view of a selected cell for each strain inside the droplets. Optical detection of 468 droplets is shown in Figure 3c, with a bimodal distribution consistent with the ratio of cell input: 91% (427 droplets) had a lower value and 9% (41 droplets) were above the threshold assigned to *sta6*. This *sta6* threshold point was based on a preliminary experiment that measured PMT output from the individual strains.

We recovered droplets that showed a PMT output signal above the threshold and plated them on an agar plate, which showed 100% viability as measured by all selected droplets resulting in colony formation. This suggests that the DMSO concentration used to dissolve BODIPY was nontoxic, and yielded a better survival rate than a previously established 5% survival for a FACS protocol (Terashima et al., 2015). Primers specific for *sta6* were designed to amplify the pARG7.8 insertion in the *STA6* gene of the *sta6* mutant (Zabawinski et al., 2001), whereas primers flanking the insertion site were used to identify the wild‐type *STA6* gene in CC‐406. We genotyped four colonies derived from droplets showing a high PMT output, with CC‐406 and *sta6* as controls. As expected, each of the high‐signal droplet‐derived colonies proved to contain only the mutant *sta6* allele (Figure 3d). These results indicate that the microfluidic microalgae lipid content screening scheme can discriminate *C. reinhardtii* cells showing higher lipid content.

### 2.5 Screening of EMS‐mutagenized cells for retrieving faster‐growing and higher lipid‐producing variants

**Figure 4:**
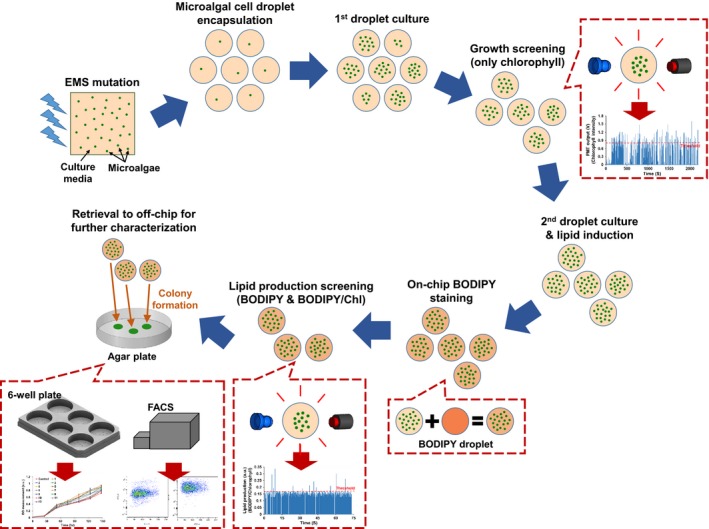
Flowchart showing the screening strategy and on‐chip steps used in this work

Following the demonstrations that the platform can detect and sort cells showing either differential growth or lipid content, it was configured to assay these two parameters in series. Figure 4 illustrates the strategy, beginning with encapsulation of EMS‐treated microalgal cells in droplets, followed by screening for faster growth, lipid induction and screening for lipid content, and finally sorting, followed by off‐chip validation.

Prior to screening the EMS‐mutated population, growth of the parental strain CC‐406 was measured in 30%–100% nitrogen to identify a condition that would both support 1.5 days of in‐droplet culture for accurate assessment of relative growth rates between clones, and also allow a rapid switch to a nitrogen‐starved environment to induce lipid accumulation (Fig. S9). Although 30% nitrogen was adequate for the analysis of lipid content (Figure 3; Fig. S8), this concentration was insufficient to identify differential growth because cell division of control CC‐406 cells had already ceased after 1.5 days of culture. Under 50% and 70% nitrogen, however, droplets support a higher cell density, which allows for identifying fast‐growing variants (Fig. S9). Therefore, 50% nitrogen was used to screen EMS‐mutated cells, in which the nitrogen levels inside the droplets will be depleted between 1.5 and 2.5 days of culture and thus initiate lipid accumulation.

**Figure 5:**
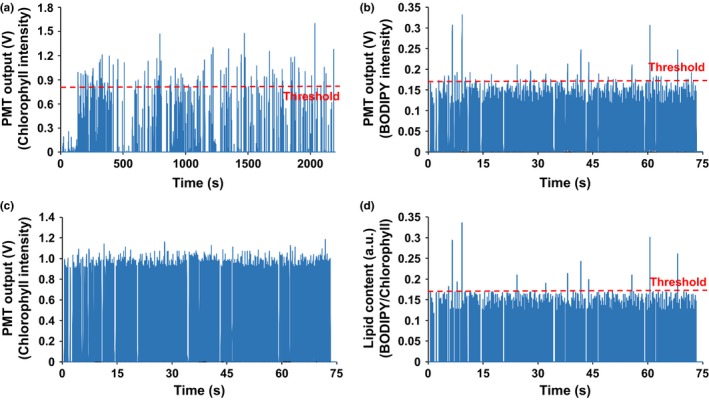
Screening of an EMS‐mutated cell population to select strains exhibiting faster growth and higher lipid content. (a) Chlorophyll autofluorescence screening after 1.5 days of culture to sort droplets showing above‐threshold growth. (b–d) Lipid content screening after an additional 2.5 days of culture. The ratio of BODIPY to chlorophyll autofluorescence in (d) was analyzed by normalizing BODIPY peaks (b) to the corresponding chlorophyll peaks (c). Droplets showing both higher signal in BODIPY fluorescence (threshold: 0.17 V) and the ratio of BODIPY to chlorophyll fluorescence (threshold: 0.175) were sorted for further off‐chip validation

Following EMS mutagenesis, approximately 200,000 individual cells were encapsulated into droplets. After 1.5 days of culture, chlorophyll autofluorescence screening was carried out, resulting in 314 droplets, which showed higher PMT signals than the threshold (Figure 5a), and thus, these droplets have a higher number of cells, presumably indicating faster growth. The sorting threshold of 0.8 V was set based on the intensity from non‐EMS‐treated CC‐406 cells in droplets (Fig. S10), which captures the top 2% of the cells in terms of their chlorophyll autofluorescence intensity. This means that any cells that show growth rate higher than that of the top 2% of the CC‐406 cells will be collected (Fig. S10). The 314 collected droplets were then cultured for an additional 2.5 days to reach stationary growth phase caused by nitrogen deprivation, thus leading to lipid accumulation. Following on‐chip BODIPY staining, 12 droplets that showed both higher BODIPY fluorescence (Figure 5b) and higher BODIPY to chlorophyll fluorescence intensity ratio (Figure 5d, which shows peaks in Figure 5b normalized to the corresponding peaks in Figure 5c) were collected and moved to an off‐chip reservoir. Normalization was used as some droplets collected from the growth screening steps had higher cell numbers than others, which can result in false‐positive hits if BODIPY fluorescence intensity alone is used as the sorting criterion. The threshold for the combined screening was set to a value corresponding to the top 2% of BODIPY to chlorophyll fluorescence intensity ratio of that coming from the control (Fig. S11).

**Figure 6:**
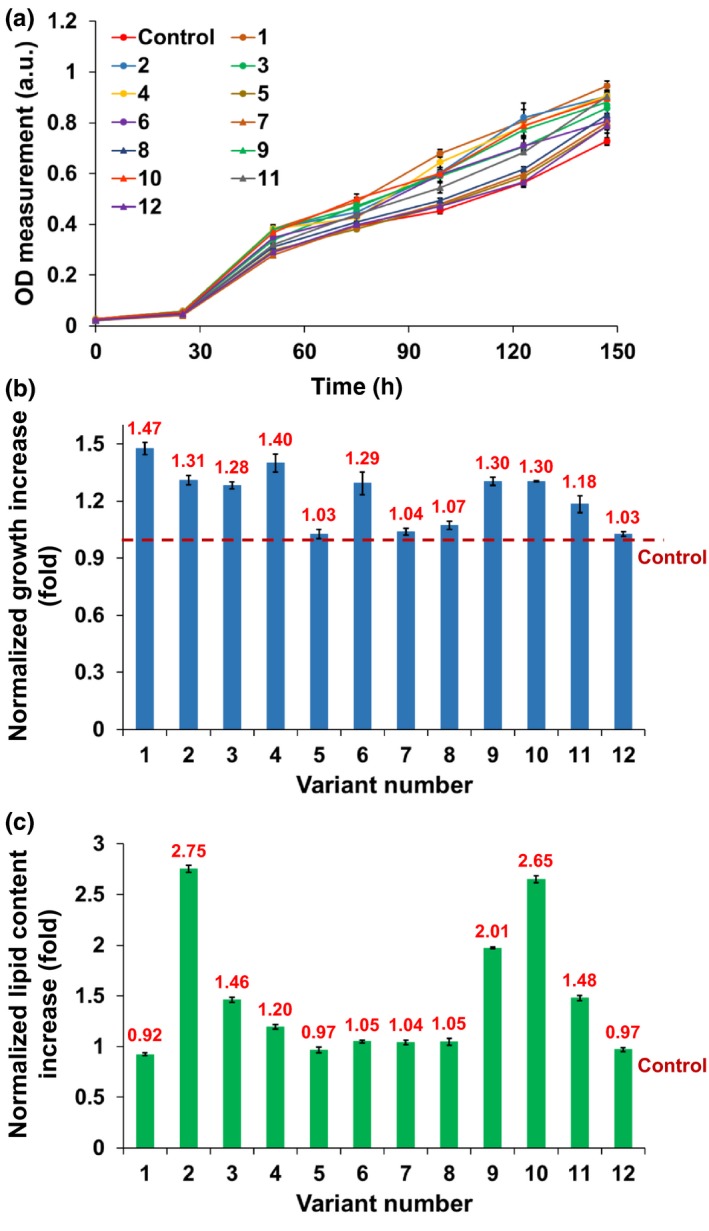
Off‐chip characterization of growth and lipid content characteristics of the 12 selected variants using a six‐well culture plate. (a) Growth comparison of the variants to the control by tracking OD_750_ over 6 days (*n = 3*). (b) After 4 days of culture, seven variants (#1, 2, 3, 4, 6, 9, and 10) showed more than 30% higher growth than the control. Growth increase (OD_day=4_/OD_day=0_) of each variant was normalized to that of the control. (c) Comparison of lipid content measured by flow cytometry (*n = 150*). All data shown are mean ± standard error

The 12 sorted droplets were plated on antibiotic‐supplemented agar to mitigate any bacterial contamination during recovery (Kan & Pan, 2010). All plated droplets successfully formed colonies, which were then used for off‐chip validation in a conventional 4.5‐ml culture in six‐well plates containing 100% nitrogen medium. As shown in Figure 6a, each of the 12 recovered clones displayed increased growth after 4 days as compared to the parental control. Of these, five variants (#1, 2, 4, 9, and 10) displayed an increase of more than 30%, and two (#1, 4) showed more than 40% (Figure 6b). Lipid content was validated through BODIPY staining followed by flow cytometry (Fig. S12). A flow cytometry analysis was performed to quantify lipid content of all variants by measuring the ratio of BODIPY to chlorophyll autofluorescence intensities, and these values were normalized to that of the control (Figure 6c). Three variants (#2, 9, and 10) had more than two‐fold increase in lipid content, and two others (#3 and 11) had more than a 40% increase. In summary, assuming that at least a 10% improvement in growth or lipid content is of interest, a total of eight variants (potential mutants) were found among the 200,000 cells screened, with six (#2, 3, 4, 9, 10, and 11) showing both improved growth and increased lipid content, whereas two (#1 and 6) showing only improved growth.

## 3 DISCUSSION

We have developed a high‐throughput droplet microfluidics‐based screening platform that is capable of analyzing both growth rate and lipid content for a large number of single cells, whether derived from a natural population or from a mutant population. Any desirable variants can be retrieved for further analysis. Key to this are a vital on‐chip and in‐droplet lipid staining process, and an optical system that can simultaneously detect chlorophyll autofluorescence and BODIPY fluorescence from the same droplet. Here, we screened a relatively small population of 2 × 10^5^ EMS‐mutagenized cells, obtaining variants with up to a 1.47‐fold growth rate increase and 2.75‐fold higher lipid content.

Traditional microalgal mutant screening, the majority of which has been carried out for *C. reinhardtii*, typically involves either a strong selective pressure or labor‐intensive and time‐consuming procedures in the laboratory. Although brute‐force screening has been widely utilized and shown success, it has substantial limitations in the number of cells that can be examined in a single assay. For example, one of the largest plate reader‐based screens for *C. reinhardtii* lipid content was limited to 34,000 putative insertional mutants (Li et al., 2012). To increase throughput, FACS has recently been utilized, which allows for high‐throughput single‐cell resolution analysis (Terashima et al., 2015; Xie et al., 2014). These studies utilized lipid‐binding dyes such as Nile red and BODIPY with FACS to isolate and examine high lipid‐producing mutants of microalgal species, including *Nannochloropsis*,* Chlorella*, and *C. reinhardtii*.

FACS, however, also has its limitations. First, cell characteristics such as cell proliferation and physiological responses at different cell densities cannot be investigated as all cells are mixed in the same pool. In contrast, droplet microfluidics allows for cell growth or physiological responses to be measured by providing physically confined independent bioreactors, in addition to being able to measure lipid content. Second, cells can be damaged during FACS due to hydrodynamic stress, which can result in a low recovery rate after sorting. For instance, only about 5% of sorted *C. reinhardtii* mutants formed colonies after Nile red staining and FACS (Terashima et al., 2015). In contrast, we observed 100% colony formation from sorted droplets, which probably results from the mild staining condition used, the shielding effect of the droplet environment from any mechanical force, and the ability to multiply a clone of cells in a droplet, meaning that colony formation does not require viability of a single cell. These advantages of using droplets, where many cells are already replicated from a single encapsulated clone, enable all clones of interest to be recovered after the staining and sorting steps even if some cells inside droplets ultimately are not viable. Third, FACS requires relatively expensive equipment, whereas a droplet microfluidics setup can cost less than $1,000 and is very simple to operate. Furthermore, each chip can easily assay more than 1 million cells. Finally, the system is computer‐controlled and does not require full‐time monitoring.

Although the developed platform has several advantages over traditional methods and has been successfully demonstrated in our screening assay, there are several aspects that can be further improved to achieve its full potential. First, the relatively slow optical analysis throughput of 5 droplets/sec can be further increased up to at least a few hundred droplets/sec relatively easily by using a high‐speed data acquisition board and implementing downstream electric field‐based droplet sorting schemes, which have achieved a droplet sorting speed of up to 2,000 events/second in some applications (Agresti et al., 2010). Second, the current droplet culture module can be enlarged to hold more droplets. We have recently developed a droplet culture module that can provide equal culturing time for 2.3 × 10^6^ droplets through a first‐in/first‐out continuous droplet processing scheme (Dai, Kim, Guzman, Shim, & Han, 2016), and plan to adopt this scheme into the platform presented here in the future. Third, currently, off‐chip validation takes a significant amount of time due to having to culture all selected droplets in a multiwell plate, followed by growth and lipid content analysis. Including an on‐chip validation step can thus significantly reduce the manual labor needed for the off‐chip validation steps. Last, the analysis capability of the platform can be enhanced by utilizing different detection methods. For example, integration of coherent anti‐Stokes Raman spectroscopy (CARS) (Wu et al., 2011) with the developed platform can provide label‐free molecule‐specific lipid analysis without having to do on‐chip BODIPY staining. In addition to significantly simplifying the overall platform, such integration can expand the application of the platform to other areas as it can enable time‐course analysis of lipid content change or lipid composition analysis, as we have demonstrated recently (Kim et al., 2017), which is not possible when using lipid‐staining dyes.

Our proof‐of‐concept screening was carried out on a small EMS‐mutated cell population, and the results are largely limited to showing the potential of the technique. However, the five strains obtained showing the desired traits of higher growth and higher lipid content point to the potential of the platform for more thorough screens of large populations or large mutant libraries. For example, only rare strains carrying multiple desirable mutations might deliver an optimal strain profile. The ability to screen in this manner, coupled to high‐throughput sequencing of either insertional mutant tags (Li et al., 2016) or whole genomes (Gallaher, Fitz‐Gibbon, Glaesener, Pellegrini, & Merchant, 2015), opens the door to rapid strain improvement, which itself could be used as a platform for further metabolic engineering (Scaife & Smith, 2016).

Although only one species was used here, there is no limitation on the type of microalgal cells that can be used as droplets can encapsulate any shape or size of cells easily, as well as single cells or colony‐forming microalgae. Regardless of microalgal species, growth can be measured through chlorophyll autofluorescence and lipid content using BODIPY or Nile red fluorescent staining. In fact, we have utilized this platform to successfully characterize the growth profile of a green microalga *Tetraselmis suecica*, and the lipid production in the colony‐forming green microalga *Botryococcus braunii* (Kim et al., 2016). In addition, previous studies using droplet microfluidics showed that cyanobacterial and microalgal species including *C. reinhardtii, Chlorella*,* Neochloris*,* Synechocystis*, and *Dunaliella* are amenable to droplet‐based studies (Abalde‐Cela et al., 2015; Dewan, Kim, McLean, Vanapalli, & Karim, 2012; Pan et al., 2011). In conclusion, lab‐on‐chip methods are beginning to realize their potential to revolutionize genetic screens in unicellular eukaryotes.

## 4 EXPERIMENTAL PROCEDURES

### 4.1 Cell preparation

Three *Chlamydomonas reinhardtii* strains were used: a cell wall‐deficient but wild‐type‐equivalent control CC‐406 (*cw15*), a starchless mutant CC‐4333 (*cw15 arg7‐7 sta6‐1::ARG7*), and a nonphotosynthetic mutant *mcd1‐2*. Unless otherwise stated, cells were cultured in Tris–acetate–phosphate (TAP) media (Gorman & Levine, 1965; Harris, Stern, & Witman, 2009) at 22°C under continuous illumination (60 μmol photons·m^−2^·s^−1^). Cells were collected during the exponential growth phase and diluted to a concentration of 1.38 × 10^5^ cells/ml before being encapsulated into droplets. Lipid accumulation of CC‐406 and *sta6* was induced by culturing in TAP medium lacking NH_4_Cl (TAP‐N) for 2–3 days. Suspensions of each strain at 1.38 × 10^7^ cells/ml (~100 cells per droplet) were used for characterizing the on‐chip BODIPY staining process.

### 4.2 Microfabrication

The two microfluidic modules (the droplet generation/culture module and the droplet staining/analysis/sorting module) comprising the screening platform were fabricated from poly(dimethylsiloxane) (PDMS, 10:1 mixture, Sylgard 184, Dow Corning, Inc., MI) using a soft lithography technique. First, the master molds for the droplet generation/culture device (microstructure height: 160 μm) and the droplet staining/analysis/sorting device (microstructure height: 100 and 160 μm) were patterned with SU‐8 photoresist (SU‐8 2075, Microchem Inc., MA) using a conventional photolithography process. Before PDMS replication, all SU‐8 master molds were coated with the surfactant (tridecafluoro‐1,1,2,2‐tetrahydrooctyl) trichlorosilane (United Chemical Technologies, Inc., Bristol, PA) to facilitate PDMS release from the master molds. PDMS devices with a thickness of 4 mm were replicated from each master mold by pouring 24 g of PDMS prepolymer. The Cr/Cu electrodes (200 and 3000 Å thick, respectively) used for electrocoalescence‐based droplet merging were patterned on a 50.8 × 76.2 mm^2^ glass slide. A 30‐μm‐thick PDMS layer was then spin‐coated on the electrode‐patterned glass slide to protect the electrodes from subsequent hydrophobic surface coating. Through oxygen plasma treatment, the PDMS droplet generation/culture device was bonded with a bare 50.8 × 76.2 mm^2^ glass slide, and the PDMS droplet staining/analysis/sorting device was aligned and bonded with the electrode‐patterned glass slide. After assembly, both PDMS devices were coated with Aquapel (Pittsburg Glass Works, LLC) to make the microfluidic channels hydrophobic to achieve stable droplet generation.

### 4.3 Optical detection system

The compact fluorescence detection module was aligned to the optical detection chamber of the device to measure the fluorescence intensity of passing droplets, where the increase in intensity is proportional to the number of cells or accumulated lipids (Fig. S2). Excitation light from a blue LED (excitation wavelength: 460–500 nm, peak: 470 nm, NSPB310B, Nichia, Tokushima, Japan) passes through an excitation filter (ET480/40x) and then to a dichroic mirror (495DCLP). The dichroic mirror reflects the excitation light vertically, and an aspheric lens (352330‐A, Thorlabs, Inc., Newton, NJ, USA) focuses the excitation light onto the optical detection chamber of the device. Another dichroic mirror (590DCLP) underneath the first dichroic mirror (495DCLP) splits the emission light; in other words, this second dichroic mirror reflects the emission light having wavelength shorter than 590 nm (from BODIPY fluorescence) while passing the emission having wavelength longer than 590 nm (from chlorophyll autofluorescence). The shorter‐wavelength light goes through a BODIPY emission filter (ET535/50m, green emission) before reaching a PMT (H10721, Hamamatsu, Inc., Japan). The longer wavelength light goes through a chlorophyll emission filter (ET610m, red emission) before reaching another PMT. The plastic optical housing (size: 48 × 68 × 77 mm^3^) for enclosing the entire optical setup was fabricated using a 3D material printer (ULTRA, Envisiontec Inc., Dearborn, MI, USA). All filters and dichroic mirrors used are from Chroma Technologies (Brattleboro, VT, USA).

### 4.4 Operation of the droplet screening platform

Fluorinert Electronic Liquid FC‐40 oil (3M, Maplewood, MN) with 2.5% surfactant (008‐FluoroSurfactant, Ran Biotechnologies, Beverly, MA) was used as the carrier oil to generate droplets as described in section 2. After droplet culture, a flow rate of 70 μl/hr was used for the carrier oil to reflow the droplets into the droplet staining/analysis/sorting module. To provide adequate spacing between droplets, additional carrier oil was injected at a flow rate of 80 μl/hr. For BODIPY droplets, carrier oil and BODIPY flow rates of 110 and 40 μl/hr were used, respectively, which resulted in one‐to‐one matching with the microalgae droplet. Thus after droplet merging, which was achieved by applying a 200‐V AC signal to the electrocoalescence electrodes, the merged droplets passed the incubation chamber at 300 μl/hr (70 + 80 + 110 + 40 = 300), allowing for 11‐min BODIPY incubation and complete staining of cells within droplets. Before these droplets reached the optical detection chamber, additional carrier oil flow was added to further space the droplets to ensure single droplet detection and sorting. For data acquisition from the PMTs as well as controlling the overall operation, an NI data acquisition module (NI9219 DAQ, National Instruments Inc., TX) was used with a custom LabVIEW (National Instruments Inc., TX) program. If the PMT signal is higher than a set threshold, the program actuates the injection of additional carrier oil into the sorting channel (actuating pressure: 27,000 Pa, duration: 150 ms), which pushes the target droplet into a collection chamber. Otherwise, the droplets continue to flow into a waste outlet.

Once screening was complete, each droplet in the collection chamber was extracted and individually placed on an agar plate. When a droplet is exposed to air, oil begins to spread across the agar, eventually breaking the droplet, releasing the microalgal cells onto the agar plate, forming a colony upon culture. In order to mitigate potential contamination during this recovery process, a combination of three antibiotics (ampicillin, cefotaxime, and carbendazim) was added to the TAP agar plate as described previously (Kan & Pan, 2010).

### 4.5 Characterization of on‐chip BODIPY staining

BODIPY 505/515 (4,4‐difluoro‐1,3,5,7‐tetramethyl‐4‐bora‐3a,4a‐diaza‐s‐indacene) staining was optimized as described in section 2. To test cell viability and growth, after droplet merging with various concentrations of DMSO (final concentration in droplets: 0.1%, 0.2%, 0.5%, 1%, 2.5%, and 10%), each droplet (containing 100 cells as described in “Cell preparation”) was cultured in a 48‐well plate and OD measured after 10 days. Next, 0.1, 0.25, 0.5, or 1 mg/ml of BODIPY in DMSO, with 10‐minute incubation and actual mixing concentration of 1%, were tested, followed by comparing staining results to off‐chip‐stained samples. For the off‐chip‐stained sample, 1 ml of cells suspended in TAP‐N was incubated with 10 μl of 1 mg/ml BODIPY in DMSO for 20 min. The effect of BODIPY concentration was tested by analyzing BODIPY fluorescence per unit area in lipid bodies and then comparing the average to that obtained from off‐chip‐stained samples. Microscopy for BODIPY fluorescence (excitation: 460–500 nm, emission: 500–550 nm) was conducted using a Zeiss Axio Observer Z1 microscope (Carl Zeiss Micro Imaging, LLC), and all microscopic images were analyzed with NIH Image J software.

### 4.6 Growth and lipid characterization

Growth profiles were characterized as described in section 2. Droplets containing cells in 0% and 40% acetate media were maintained under 2.5% CO_2_ so that the carbon source could be diffused into them through the gas‐permeable PDMS device and carrier oil (Merkel, Bondar, Nagai, Freeman, & Pinnau, 2000). All culture experiments were carried out under continuous light of 80 μmol photons·m^−2^·s^−1^. Microscopic images were taken with a chlorophyll filter set (excitation: 460–500 nm, emission >610 nm).

### 4.7 Off‐chip PCR confirmation

For genotyping, total DNA was prepared according to Werner and Mergenhagen (1998). Primers were designed to amplify sequences specific to CC‐406, *mcd1‐2*, containing a nonsense mutation in the *MCD1* gene (Murakami, Kuehnle, & Stern, 2005), or *sta6*, containing an insertion from the pARG7.8 plasmid (Zabawinski et al., 2001). To distinguish CC‐406 from *mcd1‐2*, forward primers were designed to end at the mutation site amplifying only the wild‐type (5′‐TGCAACAGGACACCGCCA‐3′) or *mcd1‐2* (5′‐TGCAA AGGACACCGCCT‐3′) alleles, with the same reverse primer 5′‐GCTGCGACAACCCCTCCTC‐3′. To differentiate CC‐406 from *sta6*, primers were designed to be either flanking the pARG7.8 insertion site (5′‐TCCATGGGCATCTACGTCAT‐3′ and 5′‐GACATGTTCACGTCGCAGTC‐3′) to detect the WT allele, or within the pARG7.8 sequence (5′‐ACCAGTGACGAAGGCTTGAG‐3′ and 5′‐TTGAGAGCCTTCAACCCAGT‐3′) to identify *sta6*. GoTaq DNA polymerase (Promega, Madison, WI) was used in all PCR according to the protocol provided by the manufacturer.

### 4.8 EMS mutagenesis and screening

EMS mutagenesis was conducted as described by Xie et al. (2014). Briefly, CC‐406 cells were grown to an OD_750_ of 0.6, centrifuged, and resuspended in 6 ml of TAP containing 225 mM EMS. Sample tubes were wrapped with aluminum foil and incubated at room temperature for 80 min using a rocking table. After incubation, cells were harvested by centrifugation and the pellets were washed three times with 12 ml of TAP. Cells were then resuspended in 6 ml of TAP and allowed to recover in the dark for 15–18 hr before screening using the microfluidic platform. At least two control samples from parental CC‐406 cells were prepared under the same culture condition as EMS‐mutated cells and utilized to determine the selection thresholds. The EMS killing rate (80%) was calculated by comparing the number of cells between the control CC‐406 sample and EMS‐treated CC‐406 sample through Evans Blue staining.

### 4.9 Off‐chip growth and lipid content analysis

To measure off‐chip growth profiles, triple replicates were diluted to 8.4 × 10^5^ cells/ml in TAP and placed into conventional six‐well plates (Day 0, OD_750_ = 0.02). These plates were cultured under continuous illumination of 30 μmol photons·m^−2^·s^−1^. OD_750_ values were measured with a microplate reader (Epoch Microplate Spectrophotometer, BioTek, Winooski, VT, USA) where a 96‐well plate containing 200 μl of each sample was used. The number of cells in each sample was counted using a hemocytometer. Lipid content was characterized using a flow cytometer (FACSAria, BD Biosciences, San Jose, CA, USA). All samples collected from an exponential growth phase were resuspended in TAP‐N media (0% nitrogen) and diluted to a concentration of 1.38 × 10^7^ cells/ml, followed by 2‐day incubation under a light intensity of 60 μmol photons·m^−2^·s^−1^. Before flow cytometry, lipids were stained with BODIPY, where 1 ml of each sample was treated with 10 μl of BODIPY stock solution (0.5 mg/ml in DMSO), incubated for 10 min in the dark, and rinsed at least three times with fresh TAP‐N media. A 488‐nm laser was used to excite BODIPY and chlorophyll fluorescence, which were detected at 525 ± 25 nm and 695 ± 45 nm, respectively. The flow cytometer measurement was considered complete when the total number of analyzed cells reached 10,000 or analysis time reached 12 seconds. Data were analyzed by FlowJo software (TreeStar, San Carlos, CA, USA).

## AUTHOR CONTRIBUTIONS

H.S.K., S.‐C.H., S.‐I.H., H.R.T., and A.R.G. designed/fabricated/characterized the microfluidic platform, cultured/characterized microalgal growth and lipid accumulation in the platform, and analyzed the data. S.‐C.H., H.R.T., D.R.B., M.T., T.P.D., and D.B.S. provided cultured microalgal cells, other chemicals, and technical assistance. H.S.K., S.‐C.H., T.P.D., D.B.S., and A.H. conceived the idea and designed the experiments. H.S.K. and A.H. oversaw the project. All authors contributed to writing the manuscript.
