# Supplementary material for: High‐throughput droplet microfluidics screening platform for selecting fast‐growing and high lipid‐producing microalgae from a mutant library
Source: Plant Direct. 2017 Sep 27;1(3):e00011. doi: 10.1002/pld3.11 (PMC6508572; doi:10.1002/pld3.11)
Supplement: Supplementary file 1 [file PLD3-1-e00011-s001.pdf]

Electronic Supporting Information

High-Throughput Droplet Microfluidics Screening Platform for Selecting Fast-Growing and High Lipid-Producing Microalgae from a Mutant Library

Hyun Soo Kim[1, 2], Shih-Chi Hsu[3], Song-I Han[1], Hem R. Thapa[4], Adrian R. Guzman[1], Daniel R. Browne[4], Mehmet Tatli[4], Timothy P. Devarenne[4], David B. Stern[3], and Arum Han[1, 5]*

[1] Department of Electrical and Computer Engineering, Texas A&M University, College Station, Texas 77843, USA

[2] Korea Institute of Machinery and Materials, Daegu Research Center for Medical Devices and Rehabilitation, Daegu, 42994, South Korea

[3] Boyce Thompson Institute, Ithaca, New York 14853, USA

[4] Department of Biochemistry and Biophysics, Texas A&M University, College Station, Texas 77843, USA

[5] Department of Biomedical Engineering, Texas A&M University, College Station, Texas 77843, USA

[*]Correspondence should be addressed to Arum Han (arum.han@ece.tamu.edu).

**Figure S1**	Microfabricated microfluidic mutant screening platform
**Figure S2**	In-house made optical detection system integrated underneath the microfluidic mutant screening platform
**Figure S3**	Single cell encapsulation efficiency in droplets
**Figure S4**	On-chip BODIPY staining characterization
**Figure S5**	Characterization of chlorophyll autofluorescence-based in-droplet cell number quantification
**Figure S6**	On-chip droplet sorting
**Figure S7**	Growth comparison of *C. reinhardtii* CC-406 and mcd1-2 with the indicated acetate (A) concentrations
**Figure S8**	Growth comparison of *C. reinhardtii* CC-406 and sta6 cells under the indicated nitrogen (N) concentrations inside droplets.
**Figure S9**	Growth characterization of *C. reinhardtii* CC-406 with the indicated nitrogen (N) concentrations
**Figure S10**	PMT output measuring chlorophyll autofluorescence intensity (indicating growth) of *C. reinhardtii* CC-406 cells after 1.5 days of culture
**Figure S11**	Lipid content and growth measurements of *C. reinhardtii* CC-406 after 4 days of culture
**Figure S12**	Characterization of lipid content in 12 selected variants through the flow cytometry measurement
**Movie S1**	Operation principle of the high-throughput droplet microfluidics screening platform

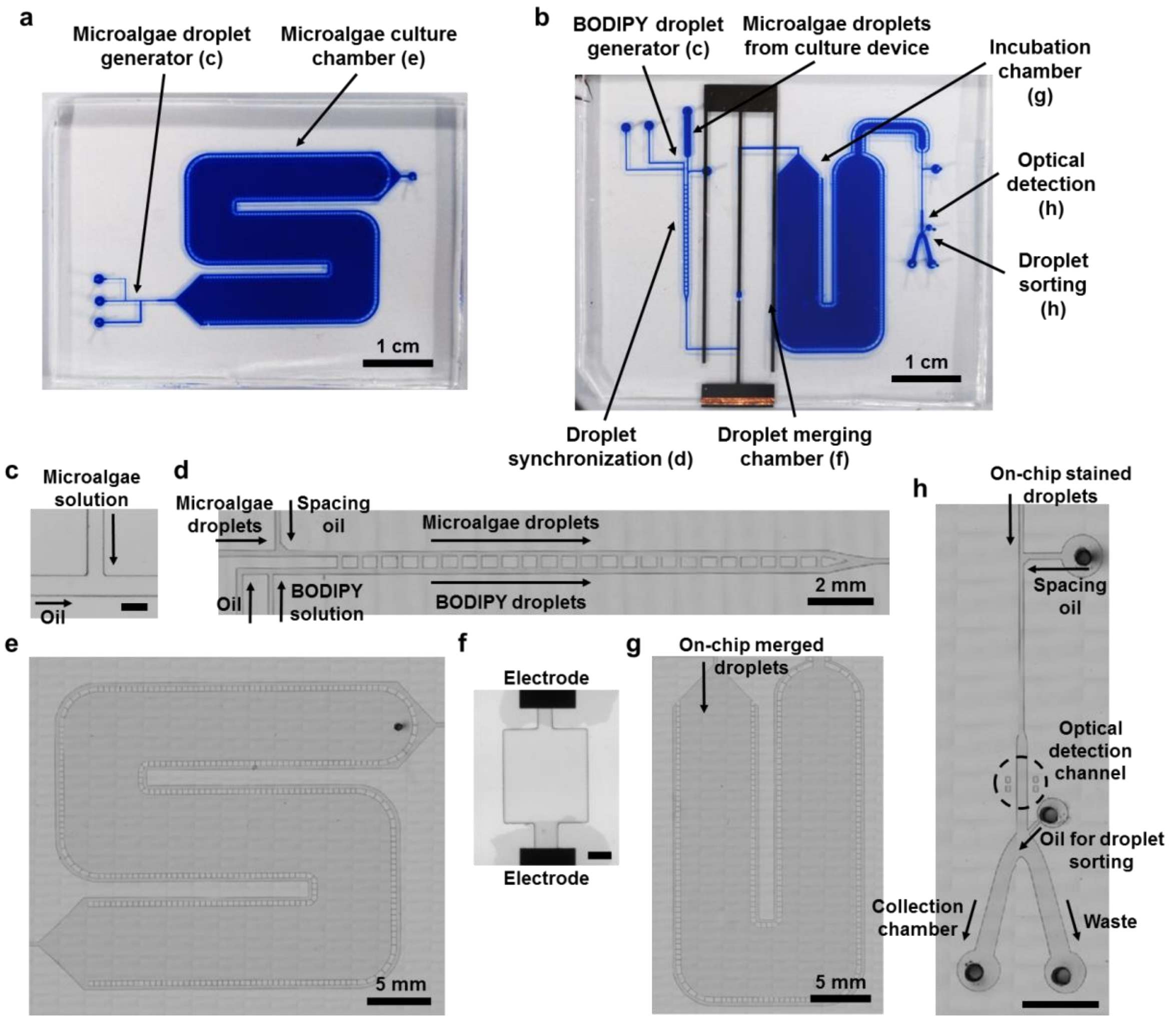

Figure S1. Microfabricated microfluidic mutant screening platform. Photographs of the two assembled micromodules: (**a**) droplet generation/culture module and (**b**) droplet staining/analysis/sorting module. Microscopic images of the (**c**) T-junction droplet generator for producing microalgae and BODIPY droplets (scale bar = 200 µm), (**d**) droplet synchronization channel where BODIPY droplets are generated and paired one-to-one with microalgae droplets, (**e**) culture chamber, (**f**) on-chip droplet merging chamber (scale bar = 200 µm), (**g**) incubation chamber, and (**h**) droplet detection channel connected to a droplet sorting channel (scale bar = 2 mm).

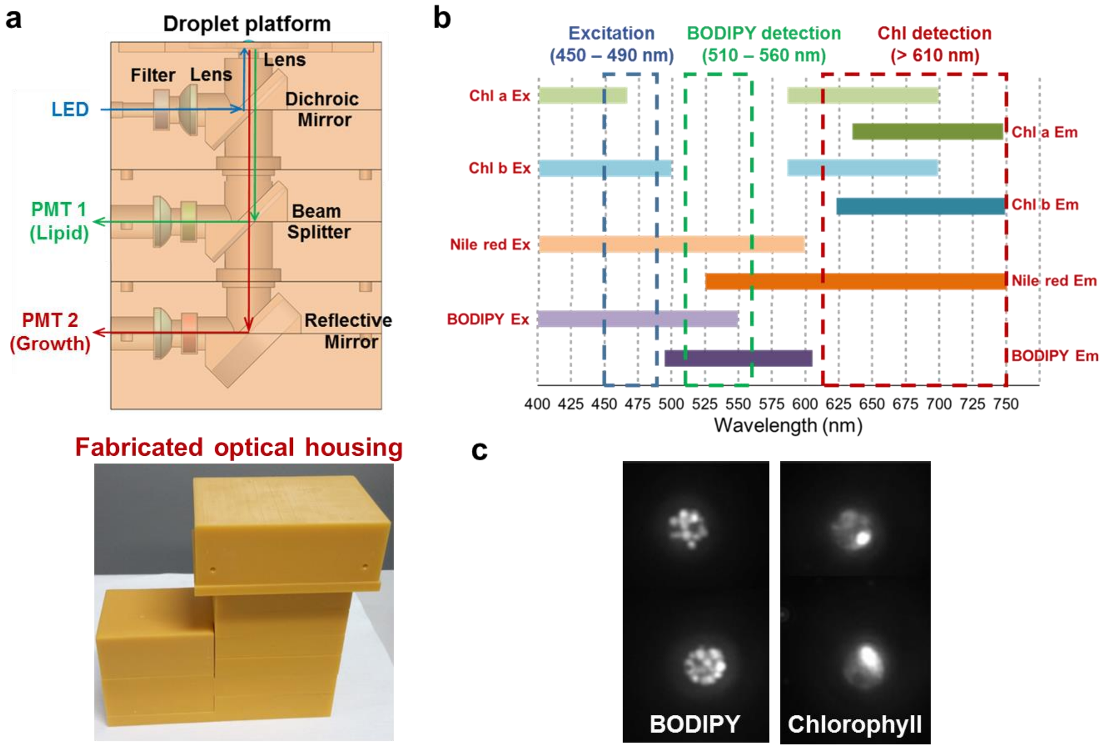

Figure S2. In-house made optical detection system integrated underneath the microfluidic mutant screening platform. (**a**) Schematic of the optical detection system capable of conducting simultaneous detection of chlorophyll autofluorescence (growth, red emission) and BODIPY (lipid, green emission) signals (size: 48 × 68 × 77 mm^3). (**b**) Excitation and emission spectrums of chlorophyll, Nile red, and BODIPY. (**c**) Micrographs of *C. reinhardtii* CC-406 cells after BODIPY staining showing no overlap between BODIPY and chlorophyll fluorescence.

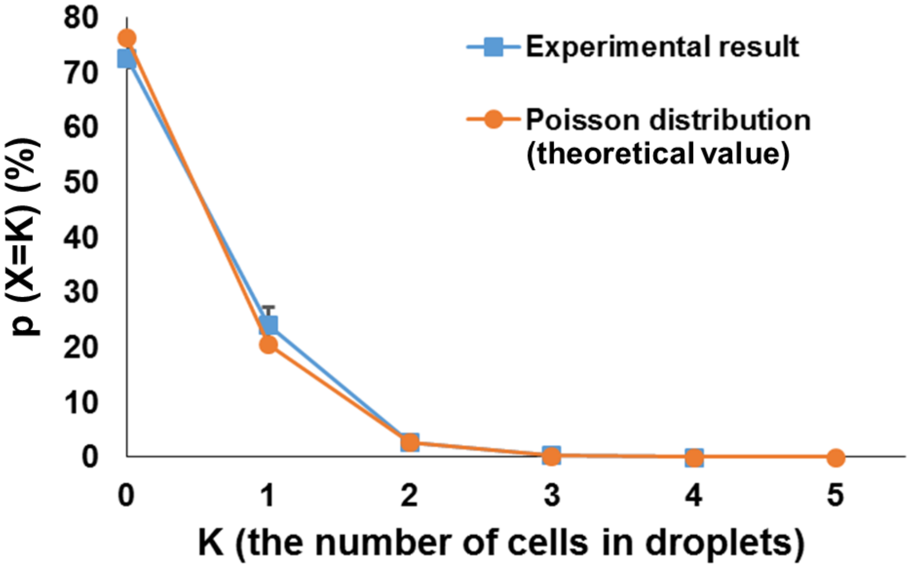

Figure S3. Single cell encapsulation efficiency in droplets. 24.1 ± 1.2% of generated droplets contained single microalga inside (blue), which follows a Poisson distribution (orange) (n = *3*).

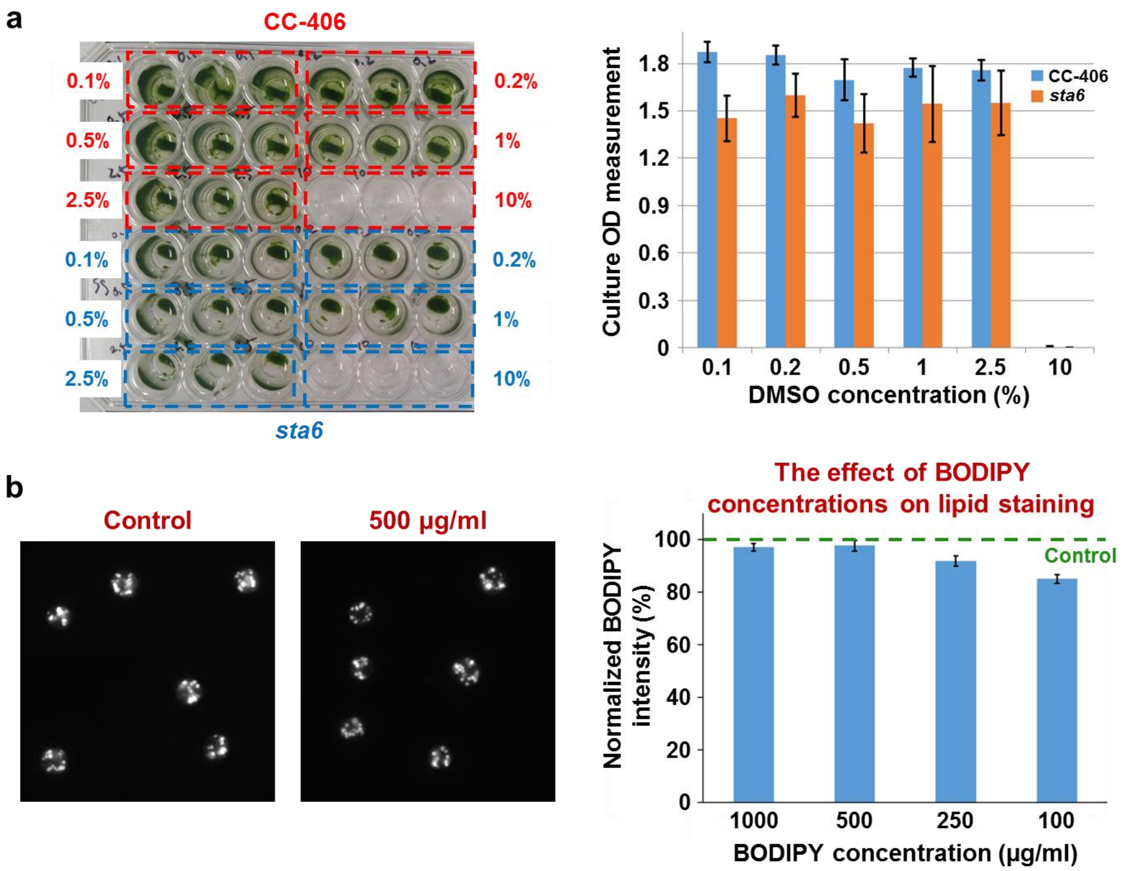

Figure S4. On-chip BODIPY staining characterization. (**a**) Cell viability test after 10-day recovery from on-chip BODIPY staining using the indicated DMSO concentrations. Single droplets containing approximately 100 cells were loaded into each culture well, one droplet per well, after the staining process (OD measurement right after droplet loading: 0). Cells stained with 2.5% or less DMSO showed growth and recovery (n = 3). (**b**) Analysis of average fluorescence intensities of lipid in *C. reinhardtii* CC-406 stained with different concentrations of BODIPY (1% (v/v) mixing concentration, 10 minute incubation). Control (off-chip stained sample) was treated with 1 mg/ml BODIPY in DMSO for 20 minutes (1% mixing concentration). BODIPY concentrations higher than 500 μg/ml showed more than 95% staining similarity compared to the control (n = 24). All data shown are mean ± standard error.

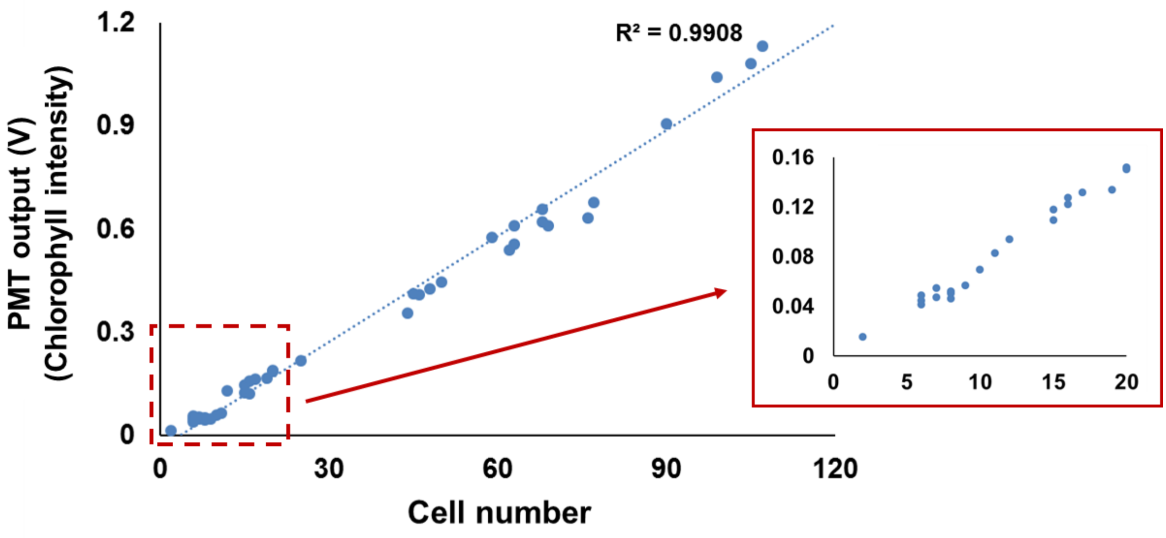

Figure S5. Characterization of chlorophyll autofluorescence-based in-droplet cell number quantification. After chlorophyll autofluorescence measurement, all detected droplets were collected sequentially in a separate channel so that the number of cells inside the droplet could be quantified through microscopy and compared with the optical measurement result. Inset shows an enlarged view of the results at low cell number per droplet, showing the capability to distinguish droplets with as low as 2 ~ 3 cell differences.

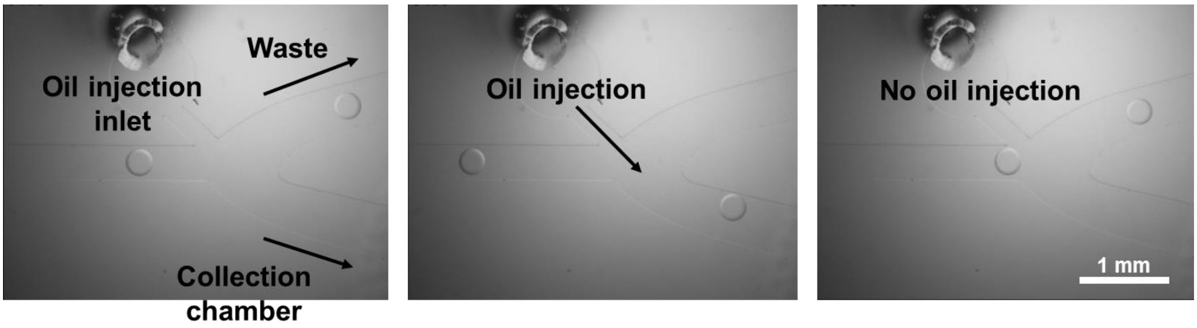

Figure S6. On-chip droplet sorting. Transient oil injection temporarily changes the flow direction of the droplets from the waste outlet to the collection chamber, allowing for a particular droplet to be selectively recovered.

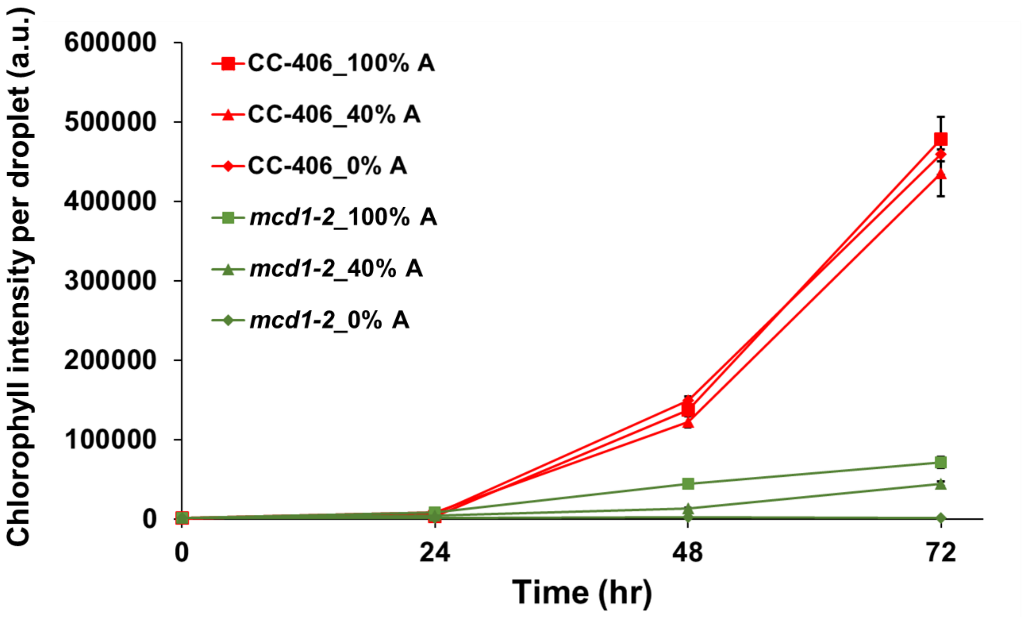

Figure S7. Growth comparison of *C. reinhardtii* CC-406 and *mcd1-2* with the indicated acetate (A) concentrations. All data shown are mean ± standard error (*n* = 17).

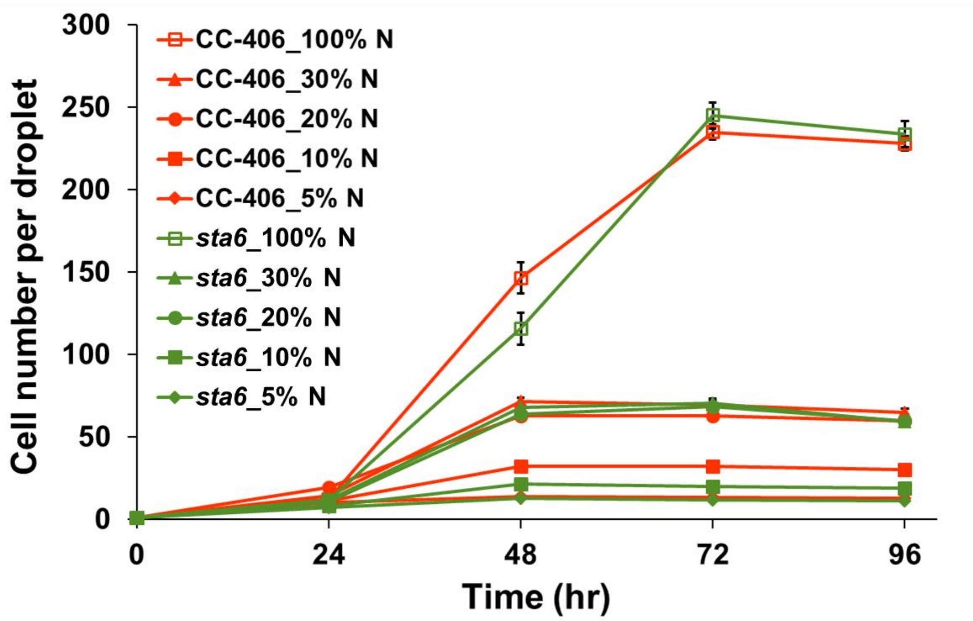

Figure S8. Growth comparison of *C. reinhardtii* CC-406 and *sta6* cells under the indicated nitrogen (N) concentrations inside droplets. Data shown are mean ± standard error (*n* = 20 droplets). At 48 hr and 30% N, average cell numbers in droplets were 70 and 71 for CC-406 and *sta6*, respectively, translating to 9.67×10^6 and 9.81×10^6 cells/ml.

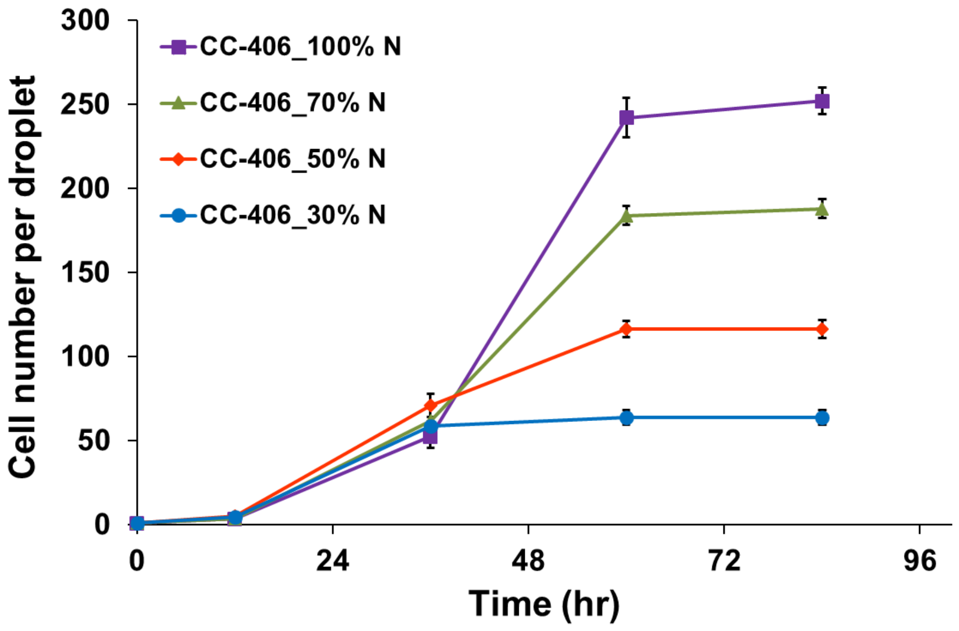

Figure S9. Growth characterization of *C. reinhardtii* CC-406 with the indicated nitrogen (N) concentrations. All data shown are mean ± standard error (n = 14).

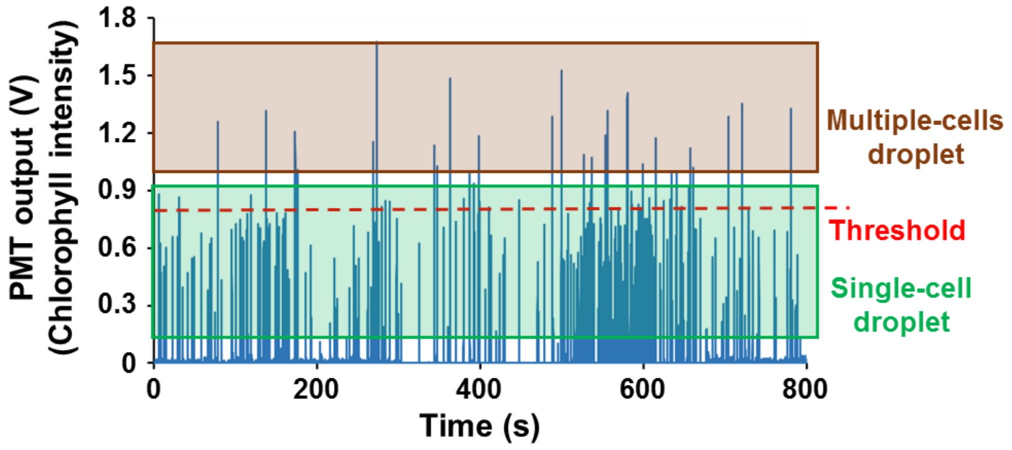

Figure S10. PMT output measuring chlorophyll autofluorescence intensity (indicating growth) of *C. reinhardtii* CC-406 cells after 1.5 days of culture. To determine the sorting threshold, signals from droplets that initially encapsulated two or more cells (brown box) were first excluded based on both the single-cell encapsulation efficiency (see Figure S3) and microscopic observation. Among peaks from the single-cell droplet region (green box), the threshold of 0.8V was set to collect EMS-mutated cells, meaning that the collected cells will have growth rate higher than that of the top 2% of CC-406 cells.

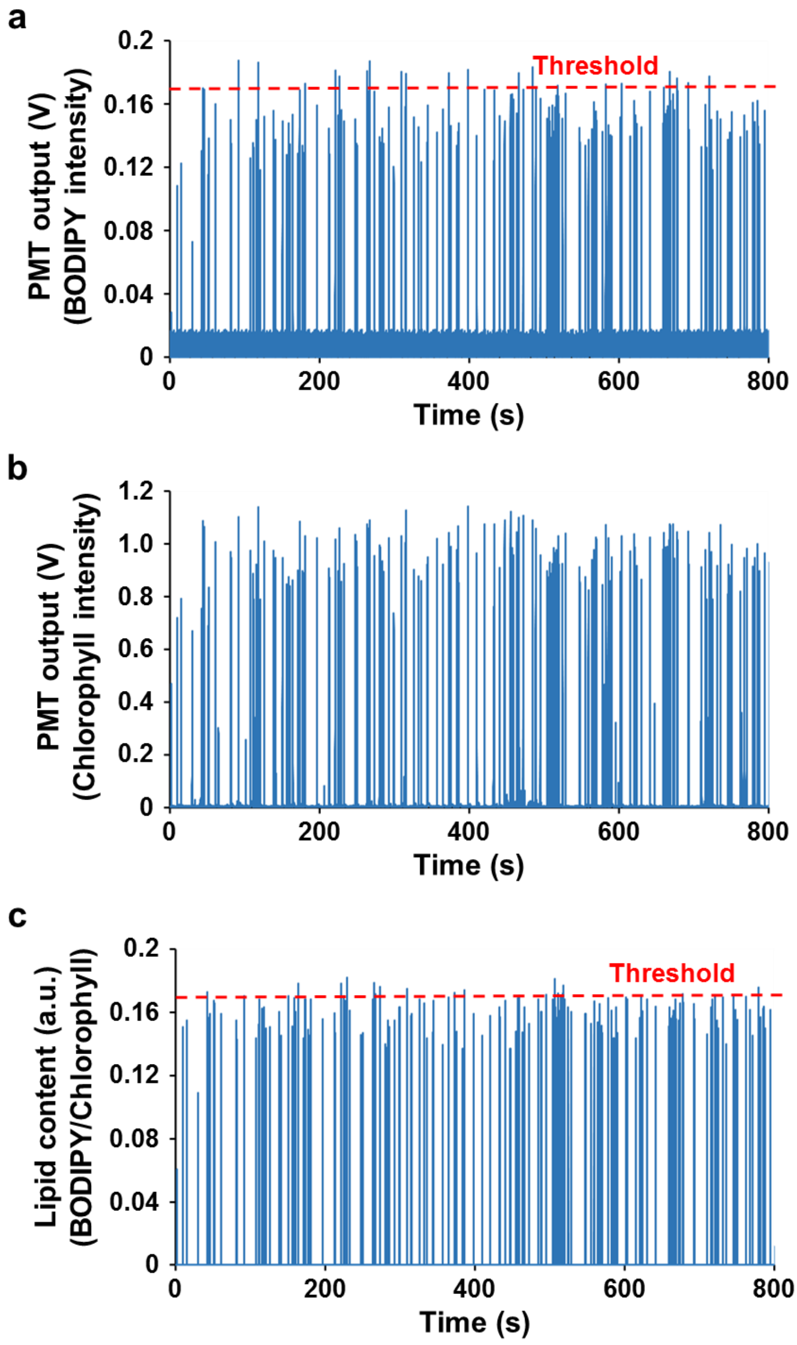

Figure S11. (**a**) Lipid content and (**b**) growth measurements of *C. reinhardtii* CC-406 after 4 days of culture. (**c**) The ratio of BODIPY to chlorophyll autofluorescence was analyzed by normalizing (**a**) BODIPY peaks to the (**b**) corresponding chlorophyll peaks.

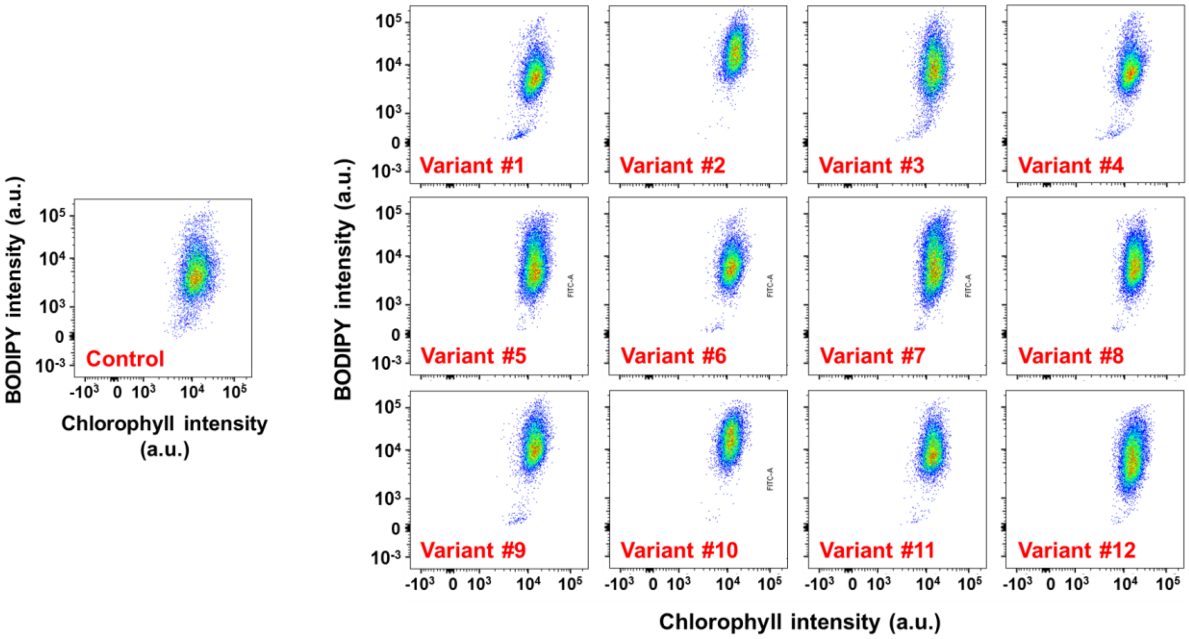

Figure S12. Characterization of lipid content in 12 selected variants through the flow cytometry measurement.
